# Therapeutic Drug Monitoring of Tacrolimus Based on Volumetric Absorptive Microsampling Technique (VAMS) in Renal Transplant Pediatric Recipients—LC-MS/MS Method Development, Hematocrit Effect Evaluation, and Clinical Application

**DOI:** 10.3390/pharmaceutics15010299

**Published:** 2023-01-16

**Authors:** Arkadiusz Kocur, Dorota Marszałek, Jacek Rubik, Agnieszka Czajkowska, Tomasz Pawiński

**Affiliations:** 1 Department of Drug Chemistry, Medical University of Warsaw, Banacha 1, 02-097 Warsaw, Poland; 2 Department of Nephrology, Kidney Transplantation and Arterial Hypertension, The Children’s Memorial Health Institute, Dzieci Polskich 20, 04-730 Warsaw, Poland; 3 Department of Biochemistry, Radioimmunology and Experimental Medicine, Pharmacokinetics Laboratory, The Children’s Memorial Health Institute, Dzieci Polskich 20, 04-730 Warsaw, Poland

**Keywords:** VAMS, tacrolimus, hematocrit effect, whole blood, kidney transplantation, pediatric population, LC-MS/MS, microsampling

## Abstract

Tacrolimus (TAC) is post-transplant pharmacotherapy’s most widely used immunosuppressant. In routine clinical practice, frequent uncomfortable venipuncture is necessary for whole-blood (WB) collection to check trough TAC levels. Volumetric absorptive microsampling (VAMS) is an alternative strategy to WB collection. In this study, we aimed to validate and develop a liquid chromatography–tandem mass spectrometry (LC-MS/MS) method for TAC quantification in WB and VAMS samples. After extraction with water and protein precipitation, the samples were directly analyzed using LC-MS/MS. Whole-blood and VAMS capillary-blood samples were collected from 50 patients treated with TAC during the follow-up visits. The cross-correlation between the developed methods was evaluated using Passing–Bablok regression and a Bland–Altman bias plot. The matrix effect (ME) and carry-over were insignificant for both scenarios. There was a high correlation between the processes and no significant clinical deviation. LC-MS/MS methods were successfully developed and validated in the 0.5–60 ng/mL calibration range. This study demonstrated and confirmed the utility of VAMS-based TAC monitoring in the pediatric population. This is the first study to directly develop and validate the VAMS LC-MS/MS method for evaluating the hematocrit effect in the pediatric population. The statistical correlation between immunochemical and VAMS-based methods was satisfactory.

## 1. Introduction

Transplantation is the best renal replacement therapy, particularly in end-stage renal disease (ESRD) patients. In this lifesaving procedure, an inefficient kidney is replaced by a perfectly healthy kidney from a living or deceased donor. The first successful pediatric renal transplant was performed in 1969 at Oregon University (Portland, OR, USA) by Goodwin, Mims, and Kaufman [1]. After transplantation, immunosuppressive therapy with drugs is necessary to prevent chronic and acute graft rejection [2].

Tacrolimus (TAC) is a calcineurin inhibitor (CNI) that constitutes the basis of post-transplant immunosuppressive pharmacotherapy in pediatric patients after solid organ transplantation (SOT), including kidney transplantation (KTX) [2]. Owing to the narrow therapeutic concentration range (NTID) and high inter- and intraindividual pharmacokinetic (PK) variability, blood TAC levels should be closely monitored to avoid under- or overdosing. Regular monitoring is necessary to balance the therapeutic range appropriately to ensure optimal graft survival [2,3,4]. Therapeutic drug monitoring (TDM) is essential for the individualization and optimization of pharmacotherapy. Christians et al. estimated that 60% of all tests performed in TDM laboratories focus on TAC determination [5]. TAC can be determined using immunochemical assays (one of the most popular methods is chemiluminescent microparticle immunoassay (CMIA) and liquid chromatography–tandem mass spectrometry (LC-MS/MS)). A total of 53% of TDM laboratories currently monitor TAC concentrations using LC-MS/MS [3,5]. Whole blood is a suitable matrix for TAC determination owing to its extensive distribution (partition) into erythrocytes (red blood cells, RBC) [4].

So far, two internal standards were used for LC-MS/MS quantification of TAC: ascomycin (ASC) and deuterated TAC (^13^C,D_2_-TAC). However, using stable isotope-labeled internal standards (SIL-IS) is still recommended worldwide. However, in the case of TAC, its structural analog (ASC) seems to be an attractive alternative. Notably, using SIL-IS, the potential matrix effect (ME), especially in LC-MS/MS techniques based on electrospray ionization (ESI), should be satisfactorily compensated [4]. On the other hand, the better chemical purity of ASC, its relatively lower price, and its higher availability in the market are substantial advantages. Currently, 60% of TDM laboratories use ASC as an IS during daily TAC determinations [5,6]. This study validated the TAC determination reference method for whole blood according to both IS.

The trough concentration (C_0_), also known as the concentration before the next dose of drug administration, is a routine parameter used in the therapeutic monitoring of TAC. The therapeutic range for TAC trough concentration (at steady state) is 5–20 ng/mL. The lower end of the range, especially in low-dose schemes, is decreased to 2–3 ng/mL and requires measurement of TAC concentrations starting from 2–3 ng/mL. Recently, it has been recommended to start with an induction dose of 100–150 mg/kg twice daily. This dose achieved C_0_ between 10 and 20 ng/mL during the first months after transplantation and between 5 and 10 ng/mL in the subsequent therapy periods [2,3]. 

There is no doubt that during immunosuppressive therapy, frequent and high-volume blood sampling is necessary to determine the overall drug exposure and dose adjustment. Therefore, microsampling techniques such as nonvolumetric dry blood spot (DBS) and recently introduced volumetric absorptive microsampling (VAMS) are gaining importance in clinical practice. In this case, a low capillary-blood volume is required for finger puncture, which is assumed to be less than 30 μL. DBS sampling is very cheap and easy to perform by patients, but on the other hand, several characteristic constraints are being observed despite over 40 years of presence in TDM. Poor sample homogeneity and volume, hematocrit, and volcano effects are the predominant problems characteristic of DBS [7]. Volume variability and hematocrit influence may be reduced using alternative methods, such as VAMS. 

The most popular tool, in this case, was the Mitra™ device introduced by Neoteryx LLC (Torrance, CA, USA) at the end of 2016. Other quantitative body fluid sampling tools are available in the diagnostic market: Capitainer (Capitainer; Solna, Sweden), hemaPen (Trajan Scientific and Medical; Ringwood, VI, Australia), and HemaXis (DBS System SA; Gland, Switzerland). These three devices are based on the DBS technique of quantitatively sampling the whole blood. Currently, VAMS is a state-of-the-art microsampling technique, particularly for the TDM. It is a high-precision volumetric technology called Mitra™ developed by Denniff and Spooner in 2014 [8].

The sampler tip is built using a hydrophilic porous polymer, which rapidly wicks biological fluids, such as whole blood, serum, urine, breast milk, cerebrospinal fluid, and saliva. The manufacturer proposed three tip volumes: 10 μL, 20 μL, and 30 μL [8,9]. The smallest sampler tips are commonly used for drug monitoring, particularly TDM. A reduction in the sample volume in TDM is only acceptable with LC-MS/MS analyte determination [10]. One lot of Mitra™ samplers was characterized by strict volume size, certified by the manufacturer, and in this study, the same series of samplers were used at an amount of 10.3 µL (<4% RSD of declared volume—10 µL). The VAMS tips absorbed capillary whole blood within a few seconds (2–4 s range) and dried rapidly at room temperature for 24 h. The drying process increases the stability of the sample and makes transport more accessible for logistical reasons. The tip may or may not be removed from the handler before the extraction. Body fluids could be collected from a finger previously disinfected and punctured using a classic lancet. The collection process was easy to use as a fingerpick for measuring blood sugar levels. Similar to DBS, this simple technique is generally intended for patient self-home sampling, which is consistent with personalized therapy rules [7]. 

This study aimed to develop and validate an LC-MS/MS method for determining TAC in capillary-blood samples using a recently introduced microsampling technique called VAMS. During the investigation, the cross-correlations between the three methods were evaluated: the new capillary VAMS microsampling with LC-MS/MS (VAMS LC-MS/MS) for classic venous sampling (WB LC-MS/MS); and routinely used CMIA. 

In our recently published review study, the currently developed methods for immunosuppressants determination (including TAC) in VAMS samples were prescribed [11]. Based on that, the main advantages of the VAMS LC-MS/MS method developed in that study are: the shortest time of sampler drying (1 h) and analytical run (5 min), simple extraction solvent (water), and finally, hematocrit evaluation and clinical cross-validation. Previously, only Kindem et al. performed a study using TAC LC-MS/MS determination in VAMS obtained from pediatric transplant recipients [12]. 

To the best of our knowledge, this is the first study in a pediatric population to evaluate the hematocrit effect (HE) according to VAMS and statistical cross-correlation between WB-LC-MS/MS, VAMS LC-MS/MS, and CMIA. Additionally, the low volume of VAMS tips used in this study, fast analytical performance, lack of hematocrit effect influence, and satisfactory accuracy of sample reanalysis seem to be strong points of study.

## 2. Materials and Methods

### 2.1. Chemical Substances, Reagents, and Internal Standards

**Figure 1:**
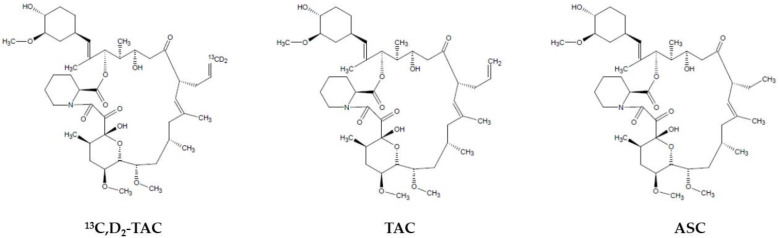
Chemical structures of the reference standard (tacrolimus, TAC) and internal standards (deuterated TAC, ^13^CD_2_TAC, and ascomycin, ASC).

The tacrolimus reference standard powder (chemical purity ≥99.00%) was obtained from Astellas Pharma Inc. (Tokyo, Japan) and Toronto Research Chemicals, Inc. (Toronto, Ontario, Canada). The internal standards, stable isotope-labeled deuterated tacrolimus-^13^CD_2_TAC (88.00% chemical purity, 86.00% isotopic purity), and the structural analog, ascomycin (ASC) (chemical purity ≥ 98.00%), were purchased from Toronto Research Chemicals Inc. (Toronto, ON, Canada) and Sigma-Aldrich (St. Louis, MO, USA), respectively. The differences in their chemical structures are shown in Figure 1. The following chromatographic solvents were used during the investigation: acetonitrile (hypergrade for LC) produced by Merck (Darmstadt, Germany) and methanol (supergradient for LC) obtained from POCH-Avantor Performance Materials (Gliwice, Poland). Additional reagents for the mobile phases, such as zinc sulfate heptahydrate (ZnSO_4_·7H_2_O, >99.00% chemical purity), ammonium fluoride (>99.99% chemical purity), and formic acid for LC-MS (>99.99% chemical purity), were acquired from Sigma-Aldrich (St. Louis, MO, USA). Water was purified and systematically obtained using a Simplicity 185 Millipore deionized system (Merck Millipore, Burlington, MA, USA). Pure LC-MS water was purchased from Sigma-Aldrich (St. Louis, MO, USA). The above reference and IS standards were stored at −20 °C in a freezer to maintain appropriate stability. Other chemical substances and reagents, such as the liquids used for mobile phase preparation, analyte extraction, and protein precipitation, were stored at room temperature or 4 °C when prepared in the experimental mixtures. To prepare calibration curves, human whole blood (WB) was freshly obtained from healthy volunteers (without TAC and other active substances influencing TAC concentration). Method validation was obtained from the Regional Centre of Blood Donation and Hemotherapy (Warsaw, Poland). Blood was stored at 4 °C and used to prepare calibration curves for one week.

The VAMS-Mitra™ 10 μL samplers for capillary-blood sampling and 96-samples autorack for sampler drying and storage cartridges were purchased from Neoteryx (Torrance, CA, USA). Vacutainer test tubes (4 mL) containing K_2_-EDTA (dipotassium salt of ethylenediamine tetra-acetic acid) as an anticoagulant for whole-blood collection, lancets, and blood collection sets were obtained from Becton Dickinson (Warsaw, Poland). Simple laboratory materials, pipettes, tips, and test tubes were purchased from Eppendorf (Hamburg, Germany). Vials with an integrated 300 μL insert and complementary caps were obtained from ThermoScientific (Waltham, MA, USA).

### 2.2. Working Solutions, Calibrators (CS), and Quality Control (QC) Samples Preparation

Individual working solutions (primary stock solutions) were prepared for TAC (100 ng/mL and 1000 ng/mL), ^13^CD_2_-TAC (1000 ng/mL), and ASC (200 ng/mL) in a methanol/water mixture (TAC; 50:50, *v*/*v*) or pure methanol (both IS) from the solid substances mentioned in Section 2.1. Working solutions of IS were diluted, such as to the concentration of ^13^CD_2_-TAC and ASC in the final sample at 15 ng/mL level. Another working solution with a lower concentration (i.e., the next stage of the working solution) was prepared by diluting the primary stock solution with a methanol/water mixture. Finally, seven working solutions were obtained for the calibration curve construction, corresponding to the appropriate calibrator concentrations: 0.5, 1.0, 2.5, 5.0, 10.0, 30.0, and 60.0 ng/mL. These calibration standards (CS) were prepared by spiking whole blood (WB) without TAC, obtained from healthy volunteers, by adding 10 μL of working solution to 50 μL of WB and immediately mixing using a self-automatic vortex (Chemland, Stargard, Poland) at room temperature. The prepared calibrators were either utilized in the WB-LC-MS/MS method or loaded into the VAMS and dried for at least one hour. Quality controls (QC) were prepared in the same manner and at four different concentrations: lower −0.75 ng/mL (LQC), medium −7.5 ng/mL (MQC_1_), 25 ng/mL (MQC_2_), and high −50 ng/mL (HQC). Complete working solutions were stored at −20 °C conditions. CS and QC samples were prepared ex tempore by spiking blood (from healthy volunteers) with TAC and IS solutions, and the remainder was discarded. 

### 2.3. Patients’ Samples and Protocol of Sampling

Whole-blood (classic venous collection) and VAMS samples (finger puncture by a lancet) for this study were obtained during regular follow-up visits between April 2022 and October 2022 from 50 pediatric renal transplant recipients treated at the Children’s Memorial Health Institute (CMHI) in Warsaw. Post-transplant pharmacotherapeutic regimens included Advagraf^®^ and Prograf^®^ (Astellas Pharma Inc., Tokyo, Japan) as once- or twice-daily TAC formulations, respectively. Additionally, patients received mycophenolic acid (MPA) CellCept^®^ (Roche AG, Basel, Switzerland) and corticosteroid drugs. Detailed demographic and clinical information about the participants is provided in Section 2.5. WB and VAMS samples were collected before the first daily TAC dose for trough concentration measurement (predose concentration). After sampling, the blood samples were stored at −20 °C, but the VAMS samplers loaded with capillary blood were stored at room temperature (RT). Samples were prepared in the next analytical step no more than a week after sampling. All the participants (or their legal guardians) provided informed consent before the study was conducted. This study was conducted in accordance with the Declaration of Helsinki, Council for International Organizations of Medical Sciences Guidelines, and Good Clinical Practice (GCP). The project was approved by the Bioethics Committee of the Children’s Memorial Health Institute in Warsaw (approval number and date: 17/KBE/2022 (20 April 2022)). Part of this study was funded by a Medical University of Warsaw “Young Researcher” grant (FW22/1/F/MB/N/22).

### 2.4. Whole-Blood Samples Treatment and Preparation for Method Validation

WB samples (total volume: 4 mL) were divided into two parts: 2 mL for routine TAC-CMIA quantification in the CMHI Pharmacokinetics Laboratory and 2 mL of WB for reference TAC quantification using the LC-MS/MS system in the Department of Drug Chemistry, Medical University of Warsaw. Simultaneously, the medical doctor collected capillary-blood samples during the follow-up visit using a VAMS Mitra™ sampling device. Samples for routine CMIA analyses were prepared using commercial kits (Abbott Laboratories, Lake Bluff, IL, USA) in a hospital medical laboratory using established proficiency-testing protocols. More information about this method is provided in Section 2.9. Samples for LC-MS/MS were prepared using the second part of the WB. In this case, 50 μL of WB was diluted with 90 μL of pure water, and 10 μL of internal standard (ASC or ^13^CD_2_-TAC) was added (simultaneously, the validation on two different IS was prepared; results can be found in Section 3). The remaining blood was stored at −20 °C. For sample purification, 400 μL of the precipitation mixture (zinc sulfate (0.1 mol/L) and acetonitrile solution, 50:50 (*v*/*v*)), were uploaded into a plastic 1.5 mL tube with the sample. Subsequently, the samples were shaken for 10 min at RT using a ThermoScientific thermoblock (ThermoScientific, Waltham, MA, USA) and inserted into a fixed-rotor MPW-375 centrifuge at 3500 rpm at 4 °C temperature conditions for 10 min. The obtained supernatant (250 μL) was transferred into glass vials and subjected to LC-MS/MS assay under the conditions detailed in Section 2.6. The complete method protocol is presented in the Figshare dataset [13,14]. 

### 2.5. VAMS Samples Treatment and Preparation for Method Validation

The VAMS samples for LC-MS/MS assays were dried for a minimum of 1 h in an autorack or the manufacturer’s original cartridges and stored in the dark at 4 °C. Subsequently, each Mitra™ sampler was placed in a plastic test tube in 150 µL of pure deionized water. Next, to extract analytes from the VAMS tips, the samples were shaken using a shaking thermoblock (ThermoScientific, Waltham, MA, USA) at RT for 1 h at a frequency of 1000 rpm. In the next step, 10 μL of IS ASC was added (after evaluation of the WB-LC-MS/MS method and the worst results of analyses with ^13^CD_2_-TAC, the authors developed and validated the VAMS method using ASC as the IS only (more details in Section 3)). A total of 150 μL precipitation mixture containing zinc sulfate (0.1 mol/L) and acetonitrile (50:50, *v*/*v*) was also added to the test tube. The samples were shaken for 10 min at room temperature using the above thermoblock. Finally, the samples were placed in a fixed-rotor MPW-375 centrifuge at 3500 rpm and 4 °C for 10 min. The obtained supernatant (250 μL) was transferred into glass vials and subjected to LC-MS/MS assay under the conditions detailed in Section 2.6. The full method protocol is presented in the Figshare dataset [13,14]. 

### 2.6. HPLC-MS/MS Equipment and Apparatus Conditions

The LC-MS/MS system used for sample analysis consisted of an 8050 triple-quadrupole MS detector (Shimadzu, Tokyo, Japan) and a set of devices for liquid chromatography. The Nexera X2 LC system (Shimadzu, Tokyo, Japan) and auxiliary equipment, namely, a binary pump for gradient flow (30AD), degasser unit (DGU-20A5R), thermostatic autosampler with vial racks (SIL-30AC), and thermostatic column oven (CTO-20AC), were coupled with the MS detector. The stationary phase was a Hypurity-C_18_ (ThermoScientific, Waltham, MA, USA) chromatographic column (50 × 2.10 mm, 3 μm) with a complementary holder with a guarded drop-in precolumn (10 × 2.10 mm, 3 μm) (ThermoScientific, Waltham, MA, USA). During the analyses, the oven temperature was set at 40 °C for the column thermostat. Additionally, a rotary six-position valve (FCV-20AH) was used to select the line between the LC system and the MS detector. The binary pump was set on the gradient flow of the mobile phase, which contained a mixture of two solutions: A and B (aqueous and organic phases, respectively). Solution A consisted of pure deionized water with an ammonium fluoride solution (final concentration in the phase was set at 2 mmol/L) supplemented with MS-purity formic acid (final concentration in the phase was set at 0.05%). Solution B consisted of an acetonitrile/methanol mixture (50:50, *v*/*v*), with the same final concentrations of ammonium fluoride and formic acid. Gradient elution during analysis in a reversed-phase chromatographic system (RP-HPLC) was set on the following 5-min LC-program schedule:

(1) Between sample injection and 0.2 min—a total volume of 90% phase A and 10% phase B;
(2) Between 1.00 and 3.10 min—a total volume of 5% of phase A and 95% of phase B;
(3) The last 2 min of a single run is the same volume proportion as the 1st stage.

The summary gradient flow was set at 0.75 mL/min during the 5 min LC program. LabSolutions software was used for multiple pair monitoring (MRM) and ion source optimization. The ammonium adducts [M+NH_4_]^+^ were observed in positive-ion mode with electrospray ionization (ESI+). The main parameters of the MS detector used in the assays are listed in Table 1. The retention time was observed at 1.50 min after sample injection for each analyte (TAC, ASC, ^13^C,D_2_-TAC). The autosampler cooler was set at 5 °C, and the samples were injected from the vials into the MS detector at a speed of 5.00 μL/sec. Only the first MRM transition was used for each analyte during the validation and calculation of the concentrations in patient samples. MRM pairs (*m/z*) were set as follows (quantitative and control pairs):

(1) TAC: 821.20 → 768.40 and 821.20 → 786.40;
(2) ^13^CD_2_-TAC: 824.56 → 771.50 and 824.56 → 789.50;
(3) ASC: 809.20 → 756.55 and 809.20 → 564.35.

**Table 1:** The MS instrument parameters were set during the analysis.

Parameter	Value
electrospray voltage	0.70 kV
detector voltage	1.82 kV
dwell time	13.0 ms
single event time	0.048 s
collision energy	22 V *
temperature of interface	250 °C
desolvation temperature	526 °C
temperature of DL ^1^	200 °C
temperature of HB ^2^	250 °C
drying gas (nitrogen) flow	5.0 L/min
heating gas (air) flow	10.0 L/min
nebulizing gas (nitrogen) flow	1.0 L/min
CID gas (argon) pressure ^3^	270 kPa
thermostatic temperature	40 °C

* Set for tacrolimus (TAC), ascomycin (ASC), and deuterated tacrolimus (^13^C,D_2_-TAC). ^1^ DL: desolvation line, ^2^ HB: heat block, ^3^ CID: collision-induced dissociation.

The injection volumes of the WB and VAMS samples were set to 1 μL and 10 μL, respectively. The other apparatus parameters and chromatographic conditions were identical for VAMS and WB assays using LC-MS/MS. Full details of the LC-MS/MS conditions are presented in the Figshare dataset [14]. 

### 2.7. Method Validation

The validation is necessary for every recently developed bioanalytical method to demonstrate its reliability and potential utility even before the first assay of patient samples. In that study, two methods were validated and evaluated: reference LC-MS/MS methods using whole blood (WB) obtained by venipuncture for two internal standards, ASC and ^13^C,D_2_-TAC, and LC-MS/MS methods based on samples collected by VAMS. EMA guidelines recommend using a stable isotope-labeled IS whenever its isotopic purity is relatively satisfactory. Based on our experience with TAC assays, we used two IS to provide the preferred one because of their better validation parameters [14,15]. To appropriate the validation process, characteristics of each method according to parameters, such as selectivity; lover limit of quantification (LLOQ); and calibration range with linearity response, accuracy, precision, matrix effect, and analyte stability testing under different conditions, are needed. Analytical validation in the case of that study was performed according to the EMA guidelines [15].

#### 2.7.1. Selectivity

During the preparation of each calibrator set for the calibration curve preparation, the selectivity of each method was individually determined using blank samples without TAC from six different sources. VAMS and whole-blood samples were prepared by spiking the matrix with the LLOQ concentration of TAC (0.5 ng/mL). For both sample sets (blank and spiked), the EMA acceptance criterion was an interference response of less than 20% of the LLOQ for TAC and less than 5% for the IS used in each TAC determination method. Two events evaluated the interference from endogenous and unknown substances according to the quantitative and control MRM pairs in the TAC and IS retention time windows. Blank and zero samples were simultaneously prepared to select the calibration run. Blank samples (*n* = 6) were prepared without IS and TAC to analyze the reactions during the entire chromatographic run. Zero samples (*n* = 6) were prepared without TAC calibrators but with IS, in addition to checking for chromatographic interference caused by IS (significant for standards with limited chemical purity, i.e., deuterated TAC).

#### 2.7.2. Carry-Over Effect

Carry-over was experimentally performed using the sequence of the HQC sample injection immediately after the blank sample without analytes. Following the EMA guidelines, the acceptance criteria for TAC are less than 20% and less than 5% for IS. 

#### 2.7.3. Calibration and Linearity

These parameters were evaluated and calculated in the range of 0.5 and 60 ng/mL based on seven concentration levels: 0.5, 1.0, 2.5, 5.0, 10.0, 30.0, and 60.0 ng/mL. The results from 10 calibration curves prepared on different days were used for linearity evaluation and calibration. In this study, linearity was verified using linear regression for 1/x weighting. 

#### 2.7.4. Accuracy and Precision

Accuracy is a validation parameter that defines the closeness of agreement between the determined concentration value and the accepted reference concentration value. This procedure should be performed using calibrators for the known analyte concentrations. Mathematically, the accuracy is expressed as the determined value/reference value percentage ratio. Precision is expressed as the degree of scattering between series samples and is defined as the percentage SD/arithmetical mean ratio. Both parameters should be evaluated within a run (also known as intraday) and between runs (also known as interday). Both parameters were calculated using the results of sample analyses at five concentration levels (LLOQ, MQC_1_, MQC_2_, HQC, and ULOQ) in a single run (*n* = 10; within-run) or different runs (*n* = 10; between-run). Acceptance values for accuracy and precision: the mean concentration should be within 15% of the reference value, whereas the LLOQ should be within 20% of the reference value.

#### 2.7.5. Autosampler Stability

The autosampler stability parameter was determined using four TAC concentrations, similar to the IS used in previously described methods. Samples were analyzed immediately after the preparation protocol was performed, and after 1, 3, and 5 days of storage in an autosampler rack, they were cooled at 5 °C. 

#### 2.7.6. Short-Term Stability

The short-term stability was evaluated by a postpreparation experiment with a protocol interrupting TAC concentrations of –7.5 and 25 ng/mL for each method. Whole-blood (TAC spiked with ^13^C,D_2_-TAC or ASC) or VAMS samples (spiked with ASC) were analyzed immediately after preparation. Similarly, the following two sets of samples for each technique were prepared and stored before and after protein precipitation at 2 h and 4 h intervals at ambient room temperature. All the procedures were repeated six times for each TAC determination method. 

#### 2.7.7. Working Solution and Samples Long-Term Stability

The results of the working-solution stability examination were reported in a previous study by Bodnar-Broniarczyk et al. [16,17]. For TAC LC-MS/MS assays in our laboratory, we used the same working solution as the reference standards and ITSD, for which the long-term stability tests are described above. Therefore, we abandoned the long-term stability tests for working solutions. 

The long-term stability of the analyzed samples was not assessed because of the specificity of therapeutic drug monitoring. TAC determination in patient samples was needed only at the time of sample collection, and the samples were not examined after long-term storage. 

#### 2.7.8. Matrix Effect

To evaluate the matrix effect (ME) as a significant problem during ESI-MS assays, a postextraction addition experiment was performed for four concentration levels of TAC, similar to the IS. Six different whole-blood samples were collected from patients not treated with TAC to determine the ME quantitatively. The experiment was conducted under established apparatus conditions using samples of reference calibrator solutions, and whole-blood samples were treated according to the postextraction determination rules. The matrix effect (ME), process efficacy (PE), and absolute recovery (AR) ratios (expressed as percentages) were calculated according to Taylor and Zhou et al. studies regarding the EMA acceptance range for ME evaluation [15,18,19]. ME, PE, and AR were calculated separately for each described method, including IS (^13^C,D_2_-TAC and ASC) using the WB method and ASC only for the VAMS method.

#### 2.7.9. Incurred Sample Reanalysis (ISR)

The incurred sample reanalysis is recommended for potential analytical differences evaluation between calibrators and samples. These differences may be caused by comedication, sample inhomogeneity, or known or unknown metabolites [15]. This validation test should be performed in separate analytical runs, on different days, for 10% of samples in case of small molecules analysis and confirmed with initial quantification result for each sample included in ISR [15]. In the case of that study, the ISR experiment was repeated twice for VAMS-LC-MS/MS and WB-LC-MS/MS methods, using ten samples obtained from patients. 

### 2.8. Statistical Analysis and Results Evaluation

LabSolutions software (version 5.98, Shimadzu, Tokyo, Japan) was used for chromatogram treatment, analyte and IS peak marking and counting, and fitting of calibration curves by 1/x weighting (linear regression line using the least-squares method to establish the best standard concentration-detector signal), calculating the calibration equation and correlation coefficient of calibration curves (R^2^), and concentration and chromatographic signal-to-noise (S/N) determination. MedCalc software (version 20.11, MedCalc, Ostend, Belgium), Statistica (version 12.5, StatSoft Inc., Kraków, Poland), and MS Excel (version 13.65, Microsoft Corporation, Redmond, WA, USA) were used to evaluate the results, including validation of the methods and statistical correlation between the method results (Passing–Bablok regression, bias estimation with the Bland–Altman procedure, and Pearson’s correlation coefficient calculation). In this study, the data are presented as arithmetic mean ± standard deviation (SD), and the coefficient of variation (CV) is presented as a percentage SD/arithmetic mean ratio. 

### 2.9. CMIA Method Details

In CMHI, as a routine method for TAC trough concentration monitoring (established range 2–30 ng/mL) in whole blood, chemiluminescent microparticle immunoassay (CMIA) was used with a commercially available, ready-to-use kit named Alinity™ (Abbott, Chicago, IL, USA). This test is a delayed one-step immunoassay for quantitatively determining immunosuppressants in human whole blood using chemiluminescent microparticle immunoassay (CMIA) technology. All samples (control and patient) were prepared manually using a precipitation reagent and centrifuged. The supernatant was decanted into tubes and placed into an Alinity *i* system. The reagents (assay diluent, antitacrolimus-coated paramagnetic microparticles, and acridinium–tacrolimus tracer) were combined to create a reaction mixture—the drug in the patient’s sample is bound to the antibodies on the microparticles. The resulting chemiluminescence reaction was measured in relative light units (RLUs). An indirect relationship existed between the amount of tacrolimus in the sample and the RLUs detected by Alinity *i* System optics. As this method is routinely used in clinical laboratories at the CMHI, validation and quality control were not performed in our study. The CMIA method has minimal metabolite implications compared with other immunochemical methods (IAs). Only metabolite II (31-O-desmethyl-TAC) and metabolite III (15-O-desmethyl-TAC) interacted in the CMIA assay. It is responsible for 15% and 3% of the parent TAC [20]. Consequently, IAs, including CMIA, could overestimate the measured TAC concentration because of their cross-reactivity with the above metabolites. More information about the reagents, sample preparation, analytical implications, and proficiency testing can be found in the CMIA Alinity Guide [21].

## 3. Results

### 3.1. Development of the Whole-Blood Method of TAC Quantification

TAC determination in whole-blood samples was performed using an LC-MS/MS system with ASC and ^13^C,D_2_-TAC as the IS. Analytical conditions were established experimentally based on the chromatographic separation data from the obtained chromatograms. The total binary flow rate was set to 0.75 mL/min, and the mobile phase was mixed in a gradient during the analytical run, consisting of water and acetonitrile/methanol mixtures with ammonium fluoride and formic acid. The oven temperature was set to 40 °C for the thermostatic chromatographic column, and the utility of the C_18_-HyPurity column as a stationary phase was also confirmed. The LC run time program, coupled with the MS detector, was set to 5 min. For each analyte (IS and TAC), the retention time was observed 1.5 min after sample injection. The experimental MS parameters were set using the LabSolutions software (Shimadzu, Tokyo, Japan). A short protocol of the above method and a description of the LC-MS/MS parameters can be found in Figshare files [13,14].

### 3.2. Validation of Whole-Blood Method of TAC Quantification

The LC-MS/MS method, based on whole-blood samples, was successfully validated in the TAC concentration range of 0.5–60 ng/mL for both the used IS: ASC and ^13^C,D_2_-TAC. 

The calibration model (weighted 1/x), summarized as the mean of the R^2^ of the 10 calibration curves for each IS, was 0.993 (y = 0.035x + 0.009) and 0.988 (y = 0.027x + 0.025) for ASC and ^13^C,D_2_-TAC, respectively. Each calibration curve consisted of seven increasing concentration levels, and the blank and zero samples were used for selectivity testing. The zero samples were prepared without TAC spiking, whereas the blank sample lacked TAC and IS addition. Both test samples were prepared by WB and subjected to all analytical protocols. The chromatographic signals of unknown or endogenous substances were lower than 15% of the lower limit of quantification (LLOQ) concentration (0.5 ng/mL), demonstrating good selectivity of the method. The lower limit of quantification (LLOQ) was set experimentally at 0.5 ng/mL based on chromatogram observations and signal-to-noise ratio (S/N) calculation for both IS. 

The accuracy and precision parameters of TAC concentrations at the LLOQ, MQC_1_, MQC_2_, and HQC were within the European Medicines Agency (EMA) acceptance ranges (accuracy within 85–115%, precision less than 15%, and less than 20% for LLOQ) [15]. These parameters were calculated according to ten intrarun and between-run TAC measurements (Table 2). 

The stability test (six replicates; four time points: initial day 0, day 1, day 3, and day 5, for both IS) during storage at 5 °C in an autosampler confirmed the satisfactory stability of the analytes in the samples (Table 3). The stability values of TAC concentrations in samples at room temperature during the stop-work procedure were also satisfactory for MQC_1_ and MQC_2_ concentrations of TAC (7.50 and 25 ng/mL, respectively). The mean stability levels set at two hours before and after extraction were calculated and are summarized in Table 4. Both IS stability values were within the EMA acceptance range for analyte stability [15]. 

Matrix effects were evaluated in the pre- and postextraction experiments. In addition, the absolute recovery (AR, %) and process efficiency (PE, %) were calculated for TAC, ASC, ^13^C,D_2_-TAC, and TAC/IS peak area ratios (Table 5). According to EMA guidelines, the ratio values are within an acceptable range [15]. 

Based on the above results, for the novel method of TAC concentration measurement in VAMS samples, ASC was chosen as a prominent IS for method development and validation because of its better results compared to ^13^C,D_2_-TAC.

TAC determination in capillary-blood samples obtained using VAMS samplers was performed using an LC-MS/MS system with ASC as the IS. The optimal drying time for VAMS after sampling or before calibration curve preparation was experimentally set to 1 h. The same precipitation mixture (zinc sulfate: acetonitrile) was used for the VAMS sample preparation. The volume of water added for blood extraction from the sampler tip was also set experimentally. It was concluded that 150 μL of pure water was sufficient to extract analytes effectively. Analytical conditions were established experimentally based on chromatographic separation data from chromatograms obtained using a previously developed WB-LC-MS/MS method. The total binary flow, mobile phase ingredients, and oven temperature were the same as in Section 2. The utility of the C_18_-HyPurity column as a stationary phase was also confirmed. The LC run time program and MS detector were set at 5 min. For each analyte (IS and TAC), the retention time was also observed as 1.5 min after sample injection. The MS parameters were set experimentally using a previously described WB-LC-MS/MS method. 

**Figure 2:**
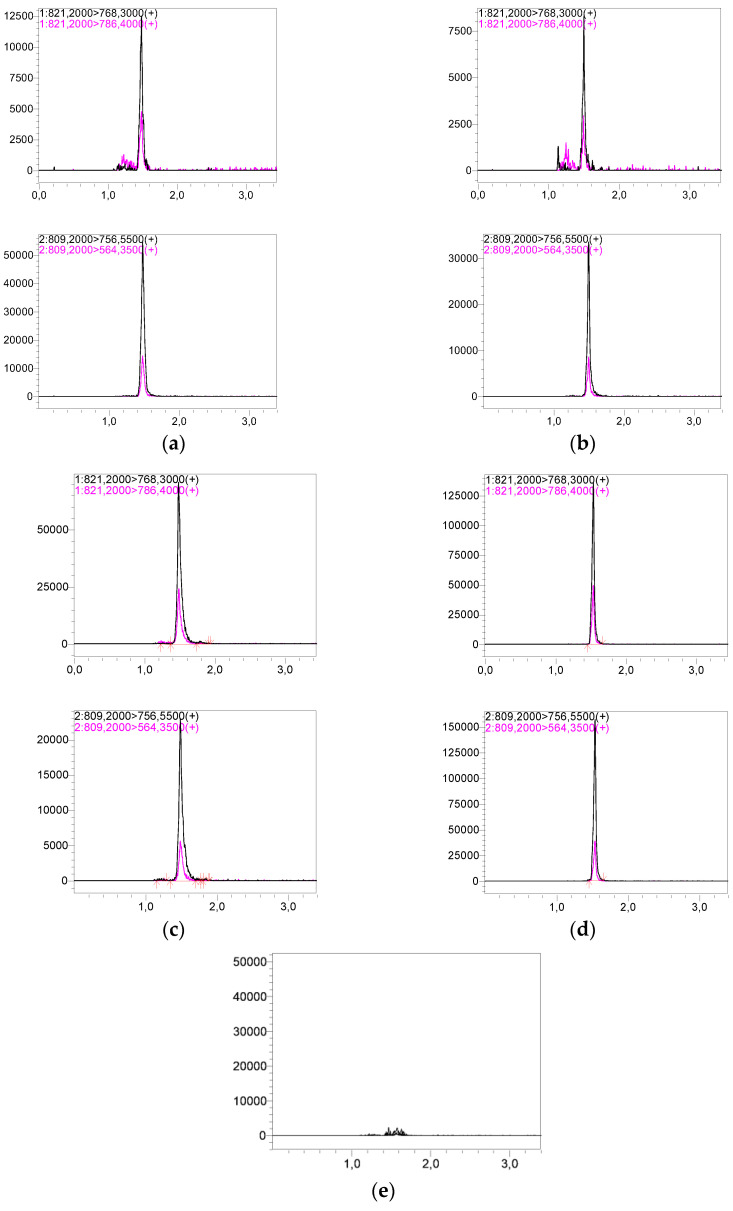
Representative chromatograms of two events (1st for TAC and 2nd for ASC respectively) for: (**a**) whole-blood patient sample—measured concentration: 3.84 ng/mL, (**b**) VAMS patient sample—measured concentration: 5.15 ng/mL, (**c**) the LLOQ—0.5 ng/mL for VAMS method, (**d**) the HQC—60 ng/mL for VAMS method, (**e**) blank sample (total ion chromatogram—TIC).

Representative chromatograms of LLOQ and HQC are shown in Figure 2. The sample preparation protocol and LC-MS/MS assay details are presented in the Figshare file [14].

### 3.3. Validation of VAMS Capillary-Blood Method of TAC Quantification

LC-MS/MS method, based on samples obtained by VAMS, was successfully validated in the TAC calibration range of 0.5–60 ng/mL, using ASC as IS. 

The methods calibration model (weighted 1/x), summarized as the mean of the R^2^ of 10 calibration curves, was 0.998 (y = 0.038x + 0.01). Each calibration curve consisted of seven concentration levels, and blank and zero samples were used for selectivity testing for whole blood. The chromatographic signals of unknown or endogenous substances were lower than 15% of the LLOQ concentration (0.5 ng/mL), demonstrating the excellent selectivity of the method. The lower limit of quantification (LLOQ) was set experimentally at 0.5 ng/mL based on chromatogram observations and signal-to-noise ratio (S/N). 

The accuracy and precision parameters of TAC concentrations at the LLOQ, MQC_1_, MQC_2_, and HQC were within the EMA acceptance ranges (accuracy within 85–115%, imprecision less than 15%, and less than 20% for LLOQ) [15]. Interestingly, the IATDMCT recommendations regarding the precision of TDM methods (CV < 10%) were also fulfilled for the examined QCs. The intra- and between-run precision and accuracy values for LLOQ, MQC_1_, MQC_2_, and HQC concentrations are presented in Table 2. 

The evaluation of the carry effect was satisfactory and fulfilled the EMA validation criteria: 1.97 ± 2.23% for TAC; 0.27 ± 0.16% for ASC [15]. 

The stability values of TAC concentrations in samples at room temperature during the stop-work procedure were also satisfactory for MQC_1_ and MQC_2_ concentrations of TAC (7.50 and 25 ng/mL, respectively). The mean stability levels set at two hours before and after extraction were calculated and are summarized in Table 3. 

The stability test (six replicates; four time points: initial day 0, day 1, day 3, and day 5, for both IS) during storage at 5 °C in an autosampler confirmed the satisfactory stability of the analytes in the samples (Table 6). 

Matrix effects were evaluated in the pre- and postextraction experiments. In addition, the absolute recovery (AR, %) and process efficiency (PE, %) were calculated for TAC, ASC, ^13^C,D_2_-TAC, and TAC/IS peak area ratios (Table 7). According to the EMA guidelines, the ratio values were within the acceptable range [15].

### 3.4. Clinical and Demographic Data

Fifty patients (33 boys and 17 girls; age range: 2.23–17.92 years old) after KTX in the Children’s Memorial Health Institute in Warsaw (CMHI) were included randomly in the study to check the utility and availability of TAC concentration measurement methods. Patients were included in the study without special requirements according to their characteristics, especially the clinical picture, comorbidities, or TAC PK data. Three samples were obtained from each patient: two 2 mL whole-blood samples (for CMIA and WB-LC-MS/MS) and a Mitra™ microsampling device. The methods developed in this study (WB-LC-MS/MS and VAMSLC-MS/MS) were used for TAC concentration measurements. A routinely performed automatic CMIA procedure was also included to compare the results for each patient. Informed consent was obtained from all renal transplant patients in the study (or their legal guardians) before venipuncture and capillary-blood collection using VAMS. Blood samples were collected from three days to 183 months after renal transplantation. TAC concentrations (in ng/mL) ranged from 3.10 to 28.13, 3.70 to 26.71, and 4.12 to 26.30, obtained by the VAMS, WB, and CMIA methods, respectively. The patient characteristics are presented in Table 8.

### 3.5. Patient Samples and Clinical Application

**Figure 3:**
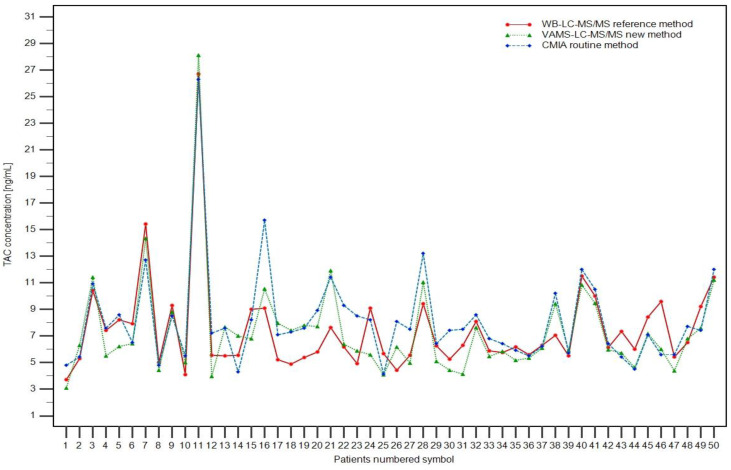
Graphical comparison of results obtained by the new VAMS LC-MS/MS method (green line with triangles), reference WB-LC-MS/MS method (red line with circles), and the routinely used automatic CMIA method (blue line with diamonds).

Successfully validated LC-MS/MS methods for TAC determination were used to analyze samples obtained from pediatric renal transplant patients. Additionally, the routinely used CMIA method was performed using the CMHI as a regular follow-up TAC trough concentration control. Maximum trough concentrations were: 28.13, 26.71, and 26.30 ng/mL for VAMS, WB-LC-MS/MS, and CMIA assays, respectively. Minimum TAC concentrations were: 3.10, 3.70, and 4.12 ng/mL for VAMS, WB-LC-MS/MS, and CMIA assays, respectively. The concentrations obtained using the three TAC methods are shown in Figure 3 for each patient. Representative chromatograms of patient samples based on WB and VAMS are shown in Figure 2. The full results of TAC determination using the described methods and hematocrit value (HCT) are published in the Figshare file [22].

### 3.6. Statistical Evaluation of Methods Correlation

**Figure 4:**
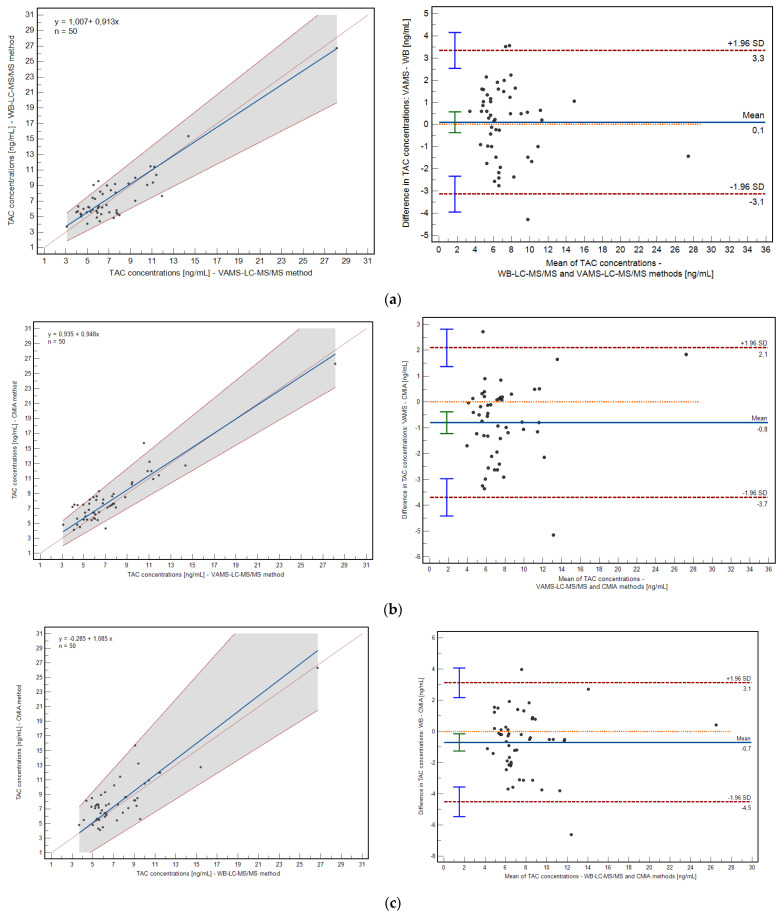
Comparison of TAC concentration results obtained from three described methods: (**a**) Passing–Bablok regression and Bland–Altman bias plot for WB-LC-MS/MS vs. VAMSLC-MS/MS; (**b**) Passing–Bablok regression and Bland–Altman bias plots for VAMSLC-MS/MS vs. CMIA; and (**c**) Passing–Bablok regression and Bland–Altman bias plot for WB-LC-MS/MS vs. CMIA.

The correlation between the developed and validated methods were evaluated using Passing–Bablok and Pearson correlation coefficient calculations. The mean bias between the methods was assessed using the Bland–Altman plot (Figure 4). 

The method based on VAMS and the WB method demonstrated a high correlation: 0.91 (*p* < 0.0001; 0.8342 to 0.9440, 95% confidence interval, CI), as well as the relationship between VAMS and the CMIA method: 0.92 (*p* < 0.0001; 0.8676 to 0.9558, 95% CI). Passing–Bablok regression confirmed high correlations for both relationships; the intercept and slope were within their statistical acceptance criteria (VAMS/WB: y = 1.006 + 0.9129x; VAMS/CMIA: y = 0.934 + 0.948x). The mean bias of the prescribed methods is shown in Figure 4. The mean difference in the case of VAMS/VB revealed 0.1046 ng/mL (−0.37 to 0.57, 95% CI), and for VAMS/CMIA comparison, it was −0.80 ng/mL (−1.22 to −0.38, 95% CI). 

The differences between the VAMS/WB and VAMS/CMIA method pairs were acceptable in the (−1.96 SD; +1.96 SD) range. Percent bias was −2.72% (−9.50 to 4.05) and −11.91% (−17.78 to −6.04) for VAMS/WB and VAMS/CMIA correlations, respectively. 

### 3.7. Evaluation of Potential Hematocrit Effect in VAMS Samples

**Figure 5:**
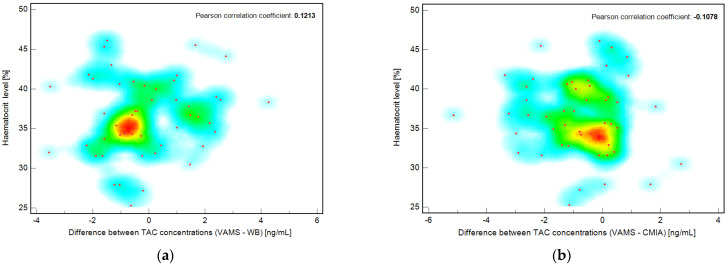
Scatter diagrams showing the poor correlation between percent hematocrit value (HCT) and differences in TAC concentrations (ng/mL) between (**a**) VAMS method results and WB method results for each patient and (**b**) VAMS method results and CMIA results for each patient. The hot map with color coding indicates the density of points according to the relationship investigation between the compared data.

To determine the effect of hematocrit level, the correlation between hematocrit level (HCT, %) and TAC concentration (from VAMS, ng/mL) was evaluated for each patient included in the study. The Pearson correlation coefficient was less than zero, confirming that no correlation was observed in this case. Additionally, the correlation coefficients were calculated for the hematocrit percentage value and the difference between the TAC concentration obtained by the VAMS and WB methods or the VAMS and CMIA methods. The results are presented as a scatter diagram with a hot map (Figure 5). The Pearson coefficients were 0.1213 (−0.1626 to 0.3866 95% CI) and −0.1078 (−0.3749 to +0.1758 95% CI) for the correlation of hematocrit value and (VAMS-WB) or (VAMS-CMIA) differences in measured TAC concentration, respectively, for each pediatric patient included in the study. To the best of our knowledge, this is the first study investigating the hematocrit effect in the pediatric population.

### 3.8. Incurred Sample Reanalysis Results 

The percent difference between the initial concentration value and the concentration level measured during the reanalysis should not be more significant than 20% of their mean for at least 67% repetitions. The differences have been calculated according to the following formula: (repeat value − initial value)/mean value of both and expressed as percent ratio [14]. In this study, all fulfilled the acceptance criteria for both validated methods. The calculation and final results of the ISR experiment can be found in the Figshare file [23].

## 4. Discussion

New methods for tacrolimus determination in whole and capillary blood were successfully established and validated. Statistical evaluation using Passing–Bablok regression and Bland–Altman plots confirmed a good correlation between the results of TAC determination in VAMS and drug concentration values obtained from wet blood. ISR experiment results confirmed satisfactory accuracy of incurred sample determination for both validated methods and limited anomalous analytical findings for reference and VAMS methods. We believe developing and validating an LC-MS/MS method for TAC determination in whole blood is necessary for sample preparation, method testing, and MS parameter optimization. During the validation of the methods based on two different IS, deuterated TAC and ASC, the structural analog of TAC was chosen as the target in VAMS LC-MS/MS method development. This step was the starting point for developing and validating the LC-MS/MS method based on VAMS and its subsequent evaluation. 

Some studies have reported that 24 h is a suitable time for drying a VAMS sampler under ambient conditions [24,25]. In our study, a reduction in the drying time to 1 h assured a high recovery of TAC, and no significant differences between the 2 h and 1 h period of VAMS drying were observed. Vethe et al. reported that 3 h of VAMS drying is satisfactory [26]. Kita et al. [27] and Koster et al. [28] reported that drying VAMS may be reduced to 2 h with no significant differences in TAC extraction accuracy.

LC-MS/MS is considered the gold standard for TAC determination and is accepted as a reference method in TDM laboratories [3,4]. Detection coupled with chromatography is characterized by excellent selectivity, sensitivity, and short analysis runtime. More than half of the TDM laboratories declared that LC-MS/MS determination of TAC was used as a reference method [5]. CMIA is used more often in smaller TDM laboratories. The primary disadvantage of immunoassays compared with LC-MS/MS is that their reliability is degraded by problems related to specific issues [3,4,5]. By contrast, validated and maintained chromatographic methods guarantee adequate and specific thorough determinations free of interference. In comparison, CMIA is easier to perform in classical laboratories because ready-to-use kits are available in the diagnostic market. In addition, commercial calibrators are readily available, which guarantees less variability among TDM laboratories. 

Performing VAMS analysis in our case took no longer than 2.5 h in summary, including the drying step and extraction; to the best of our knowledge, it is the most rapid analytical process thus far developed in the case of TAC determination. The duration of the chromatographic run was set to 5 min, although the length could be reduced by considering the retention times of the TAC and ISs. However, the longer time used in this study provided satisfactory purification of the LC-MS/MS system based on the unknown and endogenous compounds.

There are two approaches to IS loading in the VAMS methods: pre- and postsampling. The addition of the IS to the tip normalized the tip volume and satisfactorily compensated for problems with high recovery variability. However, this approach is the most difficult because the samplers require special preparation before sampling, namely, spiking with an IS solution with a specific concentration. In our case, when IS was loaded postsampling, the accuracy and precision parameters were within EMA validation ranges. Protti et al. concluded that IS should be added immediately during the analytical process [29]. In most published studies, IS was added to the solution for analyte extraction from VAMS [24,25,26, 29,30,31,32,33]. In a survey by Kita et al. [27], ASC was added after extraction from the VAMS tip, similar to our LC-MS/MS method.

Adaway and Keevil [33] reported that ASC, rather than ^13^C,D_2_-TAC, is characterized by better stability in protein precipitation solvents such as acetonitrile. Deuterated TAC may interfere with mass transitions considering the limited purity of this IS. Tron et al. used ASC as an internal standard in an LC-MS/MS assay based on TAC determination in adult patients [30]. The correlation between the reference whole-blood method and VAMS was satisfactory (Pearson correlation = 0.968) [30]. In our case, the validation results with ASC were more satisfactory than those with ^13^C,D_2_-TAC quantification in whole blood. Therefore, we used ASC as the IS for TAC determination in the VAMS samples. 

However, the manufacturer declared that the VAMS technique is independent of the hematocrit level, and its potential influence was tested in some studies. The impact of hematocrit is still unclear in some cases of using VAMS [34,35]. In a survey by Spooner et al., where the potential HCT effect was evaluated in model experiments, unequivocally, in the 20–70% range of HCT values, the visual correlation between the preferred adsorption of plasma and erythrocytes was not significant [9]. Capiau and Stove recommended simple methods to predict the HCT level in VAMS by simultaneously determining the potassium level and quantifying the primary analyte [34]. In our study, the correlation between TAC concentration differences (WB–VAMS) and HCT levels was insignificant. Consequently, the hematocrit effect was not observed in this study, and there was no relationship between HCT values and TAC blood levels, as in previous studies on adult transplant recipients [27,28,30,31,32,33]. 

Zwart et al. discussed the higher imprecision of VAMS in DBS devices [24]. However, appropriate sampling with DBS is more difficult for patients than for VAMS. The VAMS device was more attractive to patients than the DBS device. In conclusion, the VAMS technique could be used in cases of stable renal transplant recipients, as well as in cases of initiation of pharmacotherapy and clinically unstable transplant patients. In the same study, it was also evaluated that in the case of hematocrit range characteristic for transplant patients (0.20–0.60), the results of TAC determination are reliable, without significant correlation between TAC and HCT levels [24]. 

Organic solvents have been reported to be the best for hydrophobic analyte extraction from hydrophilic VAMS tips; however, in our case, water was a better extraction solvent, with satisfactory process recovery. Ye and Gao reported that a mixture of organic solvents and water was characterized by a lower elution strength, which reduced the recovery from VAMS [35]. Koster et al. [28] and Paniagua-Gonzales et al. [25] concluded that a mixture of water and methanol provides the best extraction results. According to HCT, organic solvents reduce the hematocrit effect for specific analytes. In our study, HCT ranged from 25.30 to 46.10%, and no influence was observed. 

The significant social impact and clinical potential of microsampling during immunosuppressive therapy outweigh the use of VAMS as a routine sampling method, especially in the pediatric population. Mbughuni et al. conducted a questionnaire study on satisfaction and experience with VAMS; 82% of the examinates preferred to use Mitra™ instead of venous samples [36]. After our clinical experience with VAMS blood collection, all patients included in our study and their guardians showed an interest in the VAMS technique and its potential role in the TDM of TAC, including blood collection at home. 

Previous studies on TAC determination in adult samples obtained using VAMS cannot be extrapolated to the pediatric population. Based on the PK of TAC, total body clearance (CL) in children resulted in the administration of 1.2–2-times-higher doses of TAC to ensure the efficiency of TAC action and its appropriate concentration levels at a steady state [37]. To date, excluding our study, only Kindem et al. [12] have described the clinical use of VAMS in TAC monitoring in pediatric transplant recipients, as well as in the case of home sampling based on a method developed and validated by Vethe et al. [26]. 

Our study has some limitations. It was a single-center study, and the data could not be extrapolated to the pediatric population. The impact of self-sampling by VAMS at home and the stability of samples under different conditions are required in the next step of our investigation to simulate the home sampling challenges and logistics process of sample transfer between patients and TDM laboratories. The stability of VAMS after sampling should be tested under different conditions, according to the logistic process related to obtaining samples from TDM laboratories using the classic post. Vethe et al. concluded that postal shipment does not influence the average recovery of TAC from VAMS samplers [26]. This study was performed in adult patients, and a stability examination for home-based VAMS in the pediatric population is still needed. 

## 5. Conclusions

It can be concluded that a high possibility of regular application in the therapeutic drug monitoring of tacrolimus represents an analytical method based on the VAMS. However, both have been correlated to a high degree and have consistently used one of methods; the process for individual patients is still recommended. The VAMS technique is patient-friendly and simplifies TDM; consequently, it may minimize noncompliance with therapeutic regimens because of the simplicity of blood collection and the fact that almost anyone can obtain samples precisely and quickly, anywhere, at any time without visiting a hospital. Due to the limited number of pediatric transplant centers and recent SARS-CoV-2 pandemic restrictions, this approach to blood collection seems to be an attractive alternative for young patients, their families, and all personnel involved in post-transplant pharmacotherapy. Nevertheless, the unit price of the VAMS assay is higher (around USD 3–4) than the determination of TAC with WB-LC-MS/MS or CMIA methods. Still, as a consequence, from a long-term perspective, home-based self-sampling with VAMS seems to be more beneficial for healthcare systems than regular follow-up visits. The VAMS technique provides the room to recognize PK data in the pediatric population, including pharmacokinetic profiles and a limited sample strategy (LSS), which has not yet been fully developed. 

## Figures and Tables

**Table 2:** Results of intrarun and between-run accuracy and precision examination (*n* = 10).

Parameter	LLOQ—0.50 ng/mL			MQC_1_—7.50 ng/mL			MQC_2_—25.00 ng/mL			HQC—60.00 ng/mL		
	WB- ASC	WB-dTAC	VAMS-ASC	WB- ASC	WB-dTAC	VAMS-ASC	WB- ASC	WB-dTAC	VAMS-ASC	WB-ASC	WB-dTAC	VAMS-ASC
Intrarun Accuracy and precision (*n* = 10)												
C_TAC_ (ng/mL)	0.45 ± 0.06	0.53 ± 0.08	0.52 ± 0.04	7.33 ± 0.36	7.36 ± 0.62	7.42 ± 0.28	25.48 ± 1.02	25.80 ± 1.81	25.49 ± 0.70	61.46 ± 1.31	60.36 ± 2.96	59.98 ± 1.30
Accuracy (%)	90.42	105.90	103.96	97.71	98.12	98.87	101.91	103.21	101.95	102.42	100.60	99.97
Precision (%)	12.07	15.79	8.43	4.96	8.45	3.83	4.00	7.03	2.77	2.13	4.90	2.16
Between-run Accuracy and precision (*n* = 10)												
C_TAC_ (ng/mL)	0.51 ± 0.02	0.49 ± 0.03	0.56 ± 0.03	7.60 ± 0.35	7.45 ± 0.29	7.37 ± 0.19	24.73 ± 1.70	23.95 ± 1.36	25.14 ± 0.62	60.83 ± 1.64	58.83 ± 3.13	59.93 ± 0.33
Accuracy (%)	101.22	98.39	111.62	101.33	99.18	98.24	98.90	95.82	100.58	101.38	98.05	99.90
Precision (%)	3.66	6.84	5.77	4.58	3.91	2.60	6.88	5.71	2.47	1.64	5.33	0.55

Data are expressed as mean ± standard deviation (SD; with min/max range); WB-ASC, whole-blood method with ascomycin as internal standard; WB-dTAC, whole-blood method with deuterated tacrolimus as internal standard; VAMS-ASC, VAMS method with ascomycin as internal standard.

**Table 3:** Autosampler stability of LC-MS/MS methods using WB samples.

The CONCENTRATION of TAC (ng/mL)	Calculated Concentration * (ng/mL) and Stability (%)			
	Initial Day	after 24 h	after Three Days	after Five Days
IS: dTAC (*n* = 6)				
LLOQ (0.50)	0.56 ± 0.10; 100.00	0.51 ± 0.06; 89.91	0.46 ± 0.24; 80.83	0.40 ± 0.35; 70.44
MQC_1_ (7.50)	7.65 ± 0.98; 100.00	7.51 ± 0.75; 98.22	7.33 ± 0.66; 95.85	7.02 ± 0.49; 91.89
MQC_2_ (25.0)	25.86 ± 2.31; 100.00	25.33 ± 1.54; 97.95	25.17 ± 2.88; 97.31	24.96 ± 2.27; 96.52
HQC (60.0)	61.49 ± 2.50; 100.00	60.11 ± 6.95; 97.76	58.94 ± 4.36; 95.86	55.22 ± 4.98; 89.80
IS: ASC (*n* = 6)				
LLOQ (0.50)	0.50 ± 0.06; 100.00	0.49 ± 0.06; 99.45	0.45 ± 0.10; 90.32	0.44 ± 0.05; 89.08
MQC_1_ (7.50)	7.36 ± 0.33; 100.00	7.51 ± 0.75; 99.52	7.21 ± 0.55; 98.03	6.79 ± 0.27; 92.23
MQC_2_ (25.0)	23.83 ± 0.66; 100.00	23.43 ± 0.92; 98.32	22.30 ± 0.93; 93.58	21.51 ± 0.78; 90.26
HQC (60.0)	62.43 ± 2.56; 100.00	60.88 ± 2.30; 97.53	59.85 ± 2.11; 95.87	58.27 ± 2.65; 93.34

* Data are expressed as the mean concentration ± SD; IS, internal standard; dTAC, deuterated tacrolimus; ASC, ascomycin.

**Table 4:** Stop work test summary for all WB and VAMS LC-MS/MS methods. (*n* = 6).

TAC Concentration [ng/mL]	Calculated Concentration * (ng/mL) and Stability in RT (%)								
	WB-ASC (*n* = 6)			WB-dTAC (*n* = 6)			VAMS-ASC (*n* = 6)		
	0 h	−2 h	+2 h	0 h	−2 h	+2 h	0 h	−2 h	+2 h
MQC_1_ (7.50)	7.48 ± 0.22 100.00%	7.38 ± 0.28 98.39%	7.10 ± 0.53 94.53%	7.54 ± 0.93 100.00%	7.65 ± 1.92 101.94%	7.40 ± 0.66 98.67%	7.53 ± 0.21 100.00%	7.17 ± 0.62 94.21%	7.65 ± 0.23 103.04%
MQC_2_ (25.0)	24.79 ± 0.71 100.00%	23.00 ± 1.27 92.00%	23.02 ± 1.97 92.84%	25.06 ± 1.54 100.00%	25.44 ± 2.88 101.75%	22.14 ± 2.78 88.54%	25.50 ± 0.24 100.00%	25.37 ± 0.65 100.38%	25.32 ± 0.93 100.79%

* Data are expressed as mean ± SD (with min/max range); RT, room temperature (~24 °C), WB-ASC, whole-blood method with ascomycin as internal standard; WB-dTAC, whole-blood method with deuterated tacrolimus as internal standard; VAMS-ASC, VAMS method with ascomycin as internal standard.

**Table 5:** Results of matrix effect, process efficiency, and absolute recovery examinations (*n* = 6).

Parameter	Low QC—0.50			MQC—7.50			MQC—25.00			HQC—60.00		
	TAC	ASC	F	TAC	ASC	F	TAC	ASC	F	TAC	ASC	F
WB-dTAC * (*n* = 6)												
ME (%)	−24.69 ± 2.91	−22.88 ± 11.73	−1.06 ± 0.23	−19.71 ± 3.48	−16.26 ± 8.76	−1.07 ± 0.58	−25.01 ± 11.22	−32.88 ± 15.50	0.76 ± 0.11	−27.00 ± 15.89	−17.60 ± 7.89	0.86 ± 0.10
PE (%)	57.09 ± 2.95	58.90 ± 5.60	96.22 ± 5.26	60.29 ± 7.79	66.70 ± 12.30	91.22 ± 7.70	51.43 ± 5.59	56.43 ± 14.40	96,66 ± 1.99	62.37 ± 7.88	63.03 ± 15.80	96.44 ± 6.67
AR (%)	66.40 ± 1.30	68.90 ± 2.14	97.04 ± 2.25	55.19 ± 1.15	64.19 ± 1.41	94.72 ± 7.10	59.81 ± 2.98	61.32 ± 5.59	98,49 ± 1.74	63.28 ± 15.4	62.36 ± 9.15	100.97 ± 10.40
WB-ASC * (*n* = 6)												
ME (%)	−16.68 ± 8.34	−31.27 ± 7.17	0.92 ± 0.02	−8.45 ± 3.57	−10.32 ± 8.40	0.91 ± 0.07	−22.60 ± 16.56	−36.09 ± 18.33	0.99 ± 0.06	−25.74 ± 10.96	−11.07 ± 5.57	5.31 ± 0.36
PE (%)	56.24 ± 1.74	51.10 ± 3.74	99.91 ± 8.49	51.55 ± 5.57	55.57 ± 19.77	84.30 ± 11.49	67.51 ± 13.84	69.91 ± 8.49	97.07 ± 1.70	57.09 ± 2.95	58.90 ± 5.60	99.91 ± 8.49
AR (%)	61.10 ± 1.15	65.51± 3.51	95.31 ± 4.88	60.28 ± 5.31	63.48 ± 10.67	88.79 ± 15.23	65.31 ± 4.88	67.01 ± 5.38	97.56 ± 2.15	66.40 ± 1.30	68.90 ± 2.14	95.31 ± 4.88

* Data are presented as arithmetic mean ± standard deviation, F: factor (calculated as the ratio of TAC peak area/ASC peak area); ME: matrix effect; PE: process efficiency; AR: absolute recovery; WB: whole-blood method; dTAC: internal standard; ASC: ascomycin as internal standard; TAC: tacrolimus 3.3. Development of VAMS capillary-blood method of TAC quantification.

**Table 6:** Autosampler stability for VAMS method—ASC as IS (*n* = 6).

The Concentration of TAC [ng/mL]	Calculated Concentration * (ng/mL) and Stability (%)			
	Initial Day	after 24 h	after Three Days	after Five Days
LLOQ (0.50)	0.55 ± 0.10; 100.00	0.53 ± 0.09; 94.43	0.49 ± 0.11; 86.87	0.48 ± 0.10; 87.57
LQC (0.75)	0.72 ± 0.08; 100.00	0.68 ± 0.09; 94.24	0.64 ± 0.07; 88.71	0.60 ± 0.09; 82.96
MQC1 (7.50)	7.43 ± 0.22; 100.00	6.97 ± 0.27; 94.24	6.80 ± 0.18; 91.49	6.41 ± 0.53; 86.32
MQC2 (25.0)	26.27 ± 1.02; 100.00	24.72 ± 0.90; 94.10	23.63 ± 1.20; 89.97	23.90 ± 1.43; 91.01
HQC (60.0)	62.96 ± 1.51; 100.00	61.89 ± 1.49; 98.30	58.94 ± 1.19; 93.61	57.19 ± 1.46; 90.83

* Data are expressed as the mean concentration ± SD and stability.

**Table 7:** Results of matrix effect, process efficiency, and absolute recovery examination VAMS [*n* = 6].

Parameter	Low QC—0.50			MQC—7.50			MQC—25.00			HQC—60.00		
	TAC	ASC	F	TAC	ASC	F	TAC	ASC	F	TAC	ASC	F
ME [%]	−8.08 ± 1.56	−9.35 ± 1.70	0.99 ± 0.03	−12.29 ± 5.34	−17.03 ± 7.62	0.86 ± 0.07	−12.21 ± 6.70	−18.27 ± 5.24	0.93 ± 0.05	−15.38 ± 2.51	−20.10 ± 7.21	0.86 ± 0.10
PE [%]	65.61 ± 3.37	68.38 ± 5.23	95.09 ± 2.40	60.73 ± 5.81	68.96 ± 10.70	87.07 ± 3.30	56.87 ± 3.84	60.41 ± 3.13	96.66 ± 1.99	61.45 ± 7.40	67.07 ± 8.47	92.04 ± 7.67
AR [%]	66.40 ± 1.30	68.64 ± 5.36	95.61 ± 3.34	61.61 ± 2.91	68.85 ± 2.76	89.72 ± 2.44	59.81 ± 2.98	58.52 ± 4.31	104.14 ± 3.13	66.97 ± 1.91	68.99 ± 3.80	97.67 ± 1.46

Data are presented as arithmetic mean ± standard deviation, F: factor (calculated as the ratio of TAC peak area/ASC peak area), ME: matrix effect, PE: process efficiency, AR: absolute recovery, ASC: ascomycin as ITSD, TAC: tacrolimus.

**Table 8:** Patient characteristics and demographic data (*n* = 50).

Variable	Patients’ Characteristics *
number of patients	50
total number of samples	150
sex [male/female]	33/17
age [years]	12.51 ± 3.87 (2.23–17.92)
body weight [kg]	45.07 ± 19.50 (10.20–97.90)
height [m]	1.43 ± 0.21 (0.81–1.80)
time after KTX [months]	37.82 ± 41.59 (0.10–183.00)
TAC daily dose [mg]	5.17 ± 3.21 (1.50–6.00)
TAC dose/body weight ratio [mg/kg]	0.13 ± 0.08 (0.03–0.30)
TAC formulation [Prograf^®^/Advagraf^®^]	42/8
HCT (hematocrit level) [%]	36.45 ± 4.83 (25.30–46.10)
creatinine value [mg/dL]	0.95 ± 0.48 (0.39–2.83)
GFR [mL/min/m^2^]	70.31 ± 19.30 (22.62–99.24)
VAMS tacrolimus concentration [ng/mL]	7.33 ± 3.84 (3.10–28.13)
WB tacrolimus concentration [ng/mL]	7.44 ± 3.58 (3.70–26.71)
CMIA tacrolimus concentration [ng/mL]	±3.61 (4.12–26.30)

* Data are expressed as mean ± SD (with min/max range); KTX, kidney transplantation; TAC, tacrolimus; GFR, glomerular filtration rate; VAMS, volumetric absorptive microsampling; WB, whole blood; CMIA, chemiluminescent microparticle immunoassay.

## Data Availability

Not applicable.
